# Supplementary material for: N6-methyladenosine RNA modification regulates photosynthesis during photodamage in plants
Source: Nat Commun. 2022 Dec 2;13:7441. doi: 10.1038/s41467-022-35146-z (PMC9718803; doi:10.1038/s41467-022-35146-z)
Supplement: Supplementary file 1 — Supplementary Information [file 41467_2022_35146_MOESM1_ESM.pdf]

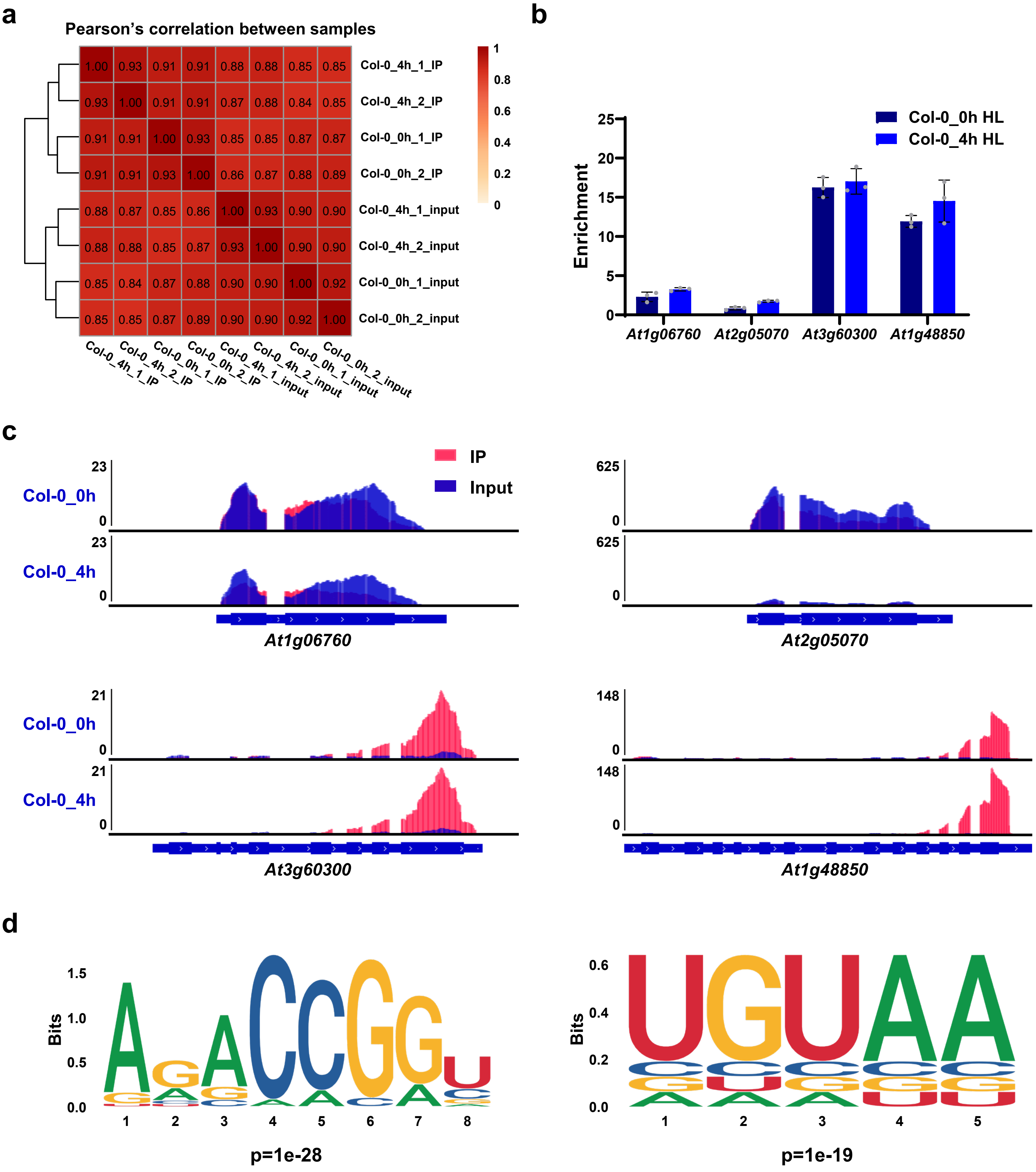


**Supplementary Figure 1. m$^6$A analysis in Arabidopsis after high light treatment (Related to Figure 1). a**, Pearson's correlation coefficients between m$^6$A profiles from two biological replicates of Col-0_0 h and Col-0_4 h. **b**, Validation of m$^6$A levels in m$^6$A-containing transcripts and negative control transcripts. Col-0 seedlings before and after a 4-h high light (HL) treatment were used for m$^6$A-IP-qPCR. The enrichment of each gene was determined by m$^6$A-IP-qPCR, first normalized by *TUB2*, and then the ratio of the abundance of the IP sample against the input sample was calculated. Values are means ± SE (n = 3 biological replicates). **c**, m$^6$A peaks of two m$^6$A-containing transcripts and two negative control transcripts in Col-0_0 h HL and Col-0_4 h HL. Blue, input reads; red, IP reads. The gene models are shown below, with thick boxes and lines representing exons and introns, respectively. **d**, RRACH (R= A/G, H = A/C/U) and URUAY (R=G>A, Y=U>A) motifs were found in most m$^6$A peaks in Col-0.

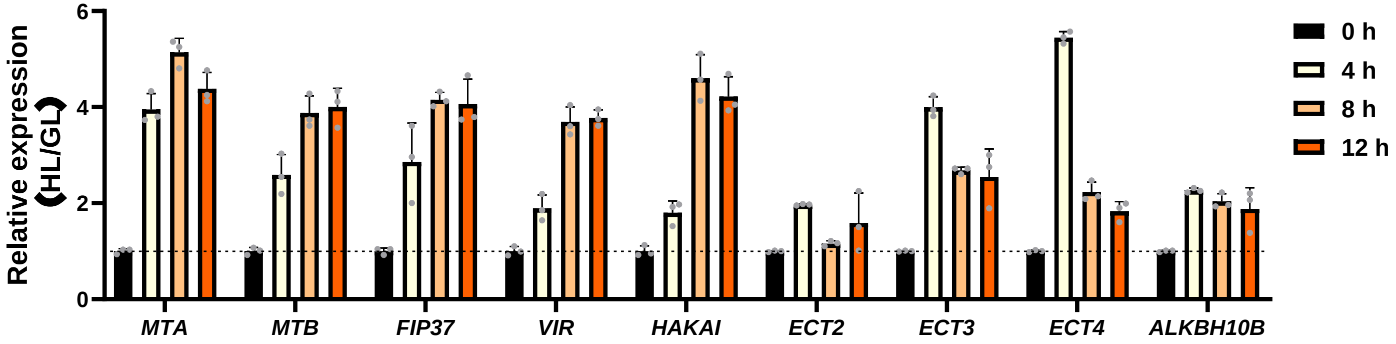


**Supplementary Figure 2. Analysis of the expression patterns of m$^6$A regulator genes in seedlings after high light treatment compared to normal growth light by qRT-PCR.** Seven-day-old Col-0 seedlings grown in a growth chamber and high light (HL) chamber for 0, 4, 8, and 12 h were collected. *UBQ10* was used as an internal control. Values are means ± SE (n = 3 biological replicates). HL, high light. GL, growth light.

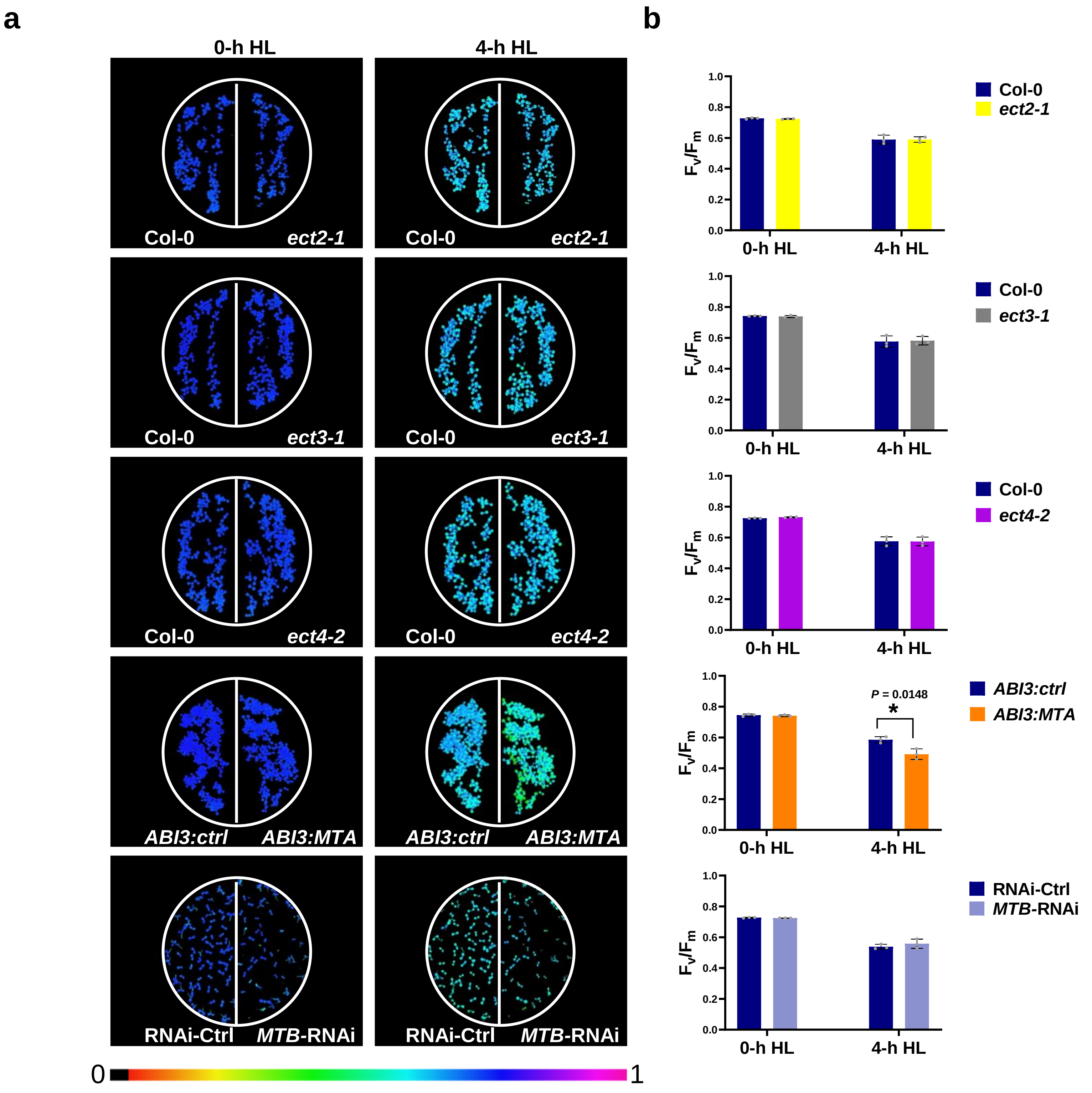


**Supplementary Figure 3. Analysis of photosynthetic activity in m⁶A regulator mutant seedlings. a,** Fluorescence images used to measure $F_v/F_m$ of seedlings grown under different light conditions. False-color images representing $F_v/F_m$ after 0- and a 4-h high light (HL) treatment in 7-day-old control and m⁶A regulator mutant seedlings, including *ect2-1*, *ect3-1*, *ect4-2*, *ABI3:MTA* and β-estradiol inducible *MTB*-RNAi, are shown. The false color ranges from black (0) to purple (1), as indicated in the scale at the bottom. For the *MTB* RNAi seedings and the corresponding control, plant material was grown on medium containing 5 μM β-estradiol. **b,** Changes in $F_v/F_m$ values for 7-day-old control and m⁶A regulator mutant seedlings under HL treatment. Values are means ± SE (n = 3 biological replicates). *, $P < 0.05$; **, $P < 0.01$, by two-sided Student's *t*-test.

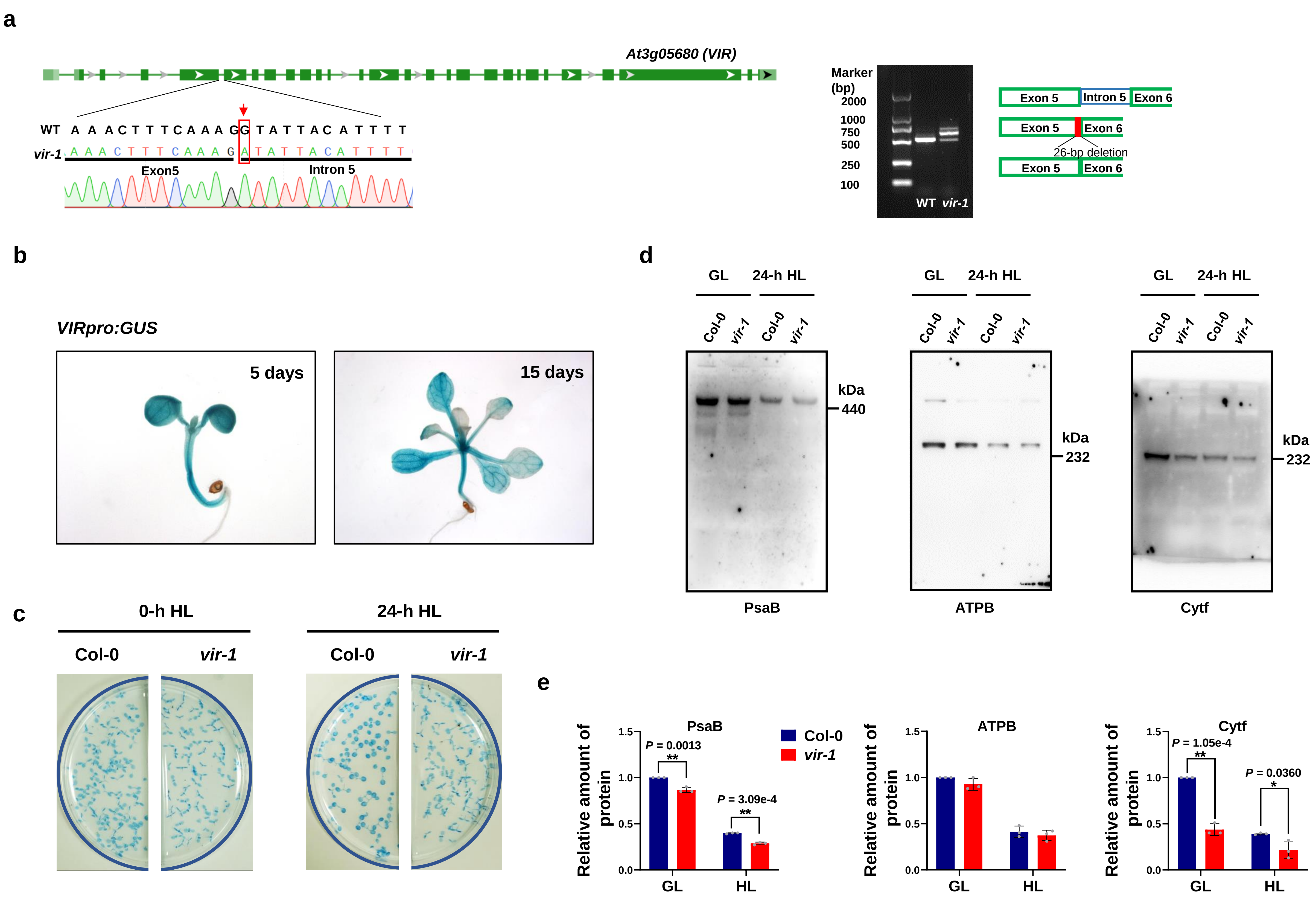


**Supplementary Figure 4. Physiological and biochemical analysis of Col-0 and *vir-1*. a**, A single nucleotide at the 5′ splice site of intron 5 of the *VIR* gene was replaced in the *vir-1* mutants, which affected splicing. RT-PCR was used to analyze the splicing pattern of *VIR* transcripts in the wild type (WT) and *vir-1* mutants. The sequencing results of the major transcripts in *vir-1* mainly include three types of products: (1) *VIR* intron 5 is retained; (2) 26 bases are missing at the 3' end of *VIR* exon 5; and (3) *VIR* is spliced correctly. **b**, GUS staining of 5- and 15-day-old *VIRpro*:*GUS* seedlings in Col-0. **c**, Trypan blue staining showing the levels of cell death in Col-0 and *vir-1* seedlings after high light (HL) treatment. **d**, BN-PAGE and immunoblot analysis of thylakoid photosynthetic complexes. Thylakoid membranes from Col-0 and *vir-1* seedlings exposed to a 0- or 72-h HL treatment were solubilized with 2% DM and separated by BN-PAGE (see Figure 3a). For BN-PAGE immunoblot analysis, the BN-PAGE gel was probed with anti-PsaB, anti-ATPB, and anti-Cyt *f* antisera. An equal amount of chlorophylls was loaded in each lane. All experiments involved three independent biological replicates, which produced similar results. **e**, Proteins immunodetected from (**d**) were quantified with Phoretix 1D Software (Phoretix International, UK). Values (mean ± SE, $n$ = 3 independent biological replicates) are given relative to protein levels of Col-0 before HL treatment. *, $P < 0.05$; **, $P < 0.01$, by two-sided Student's $t$-test.

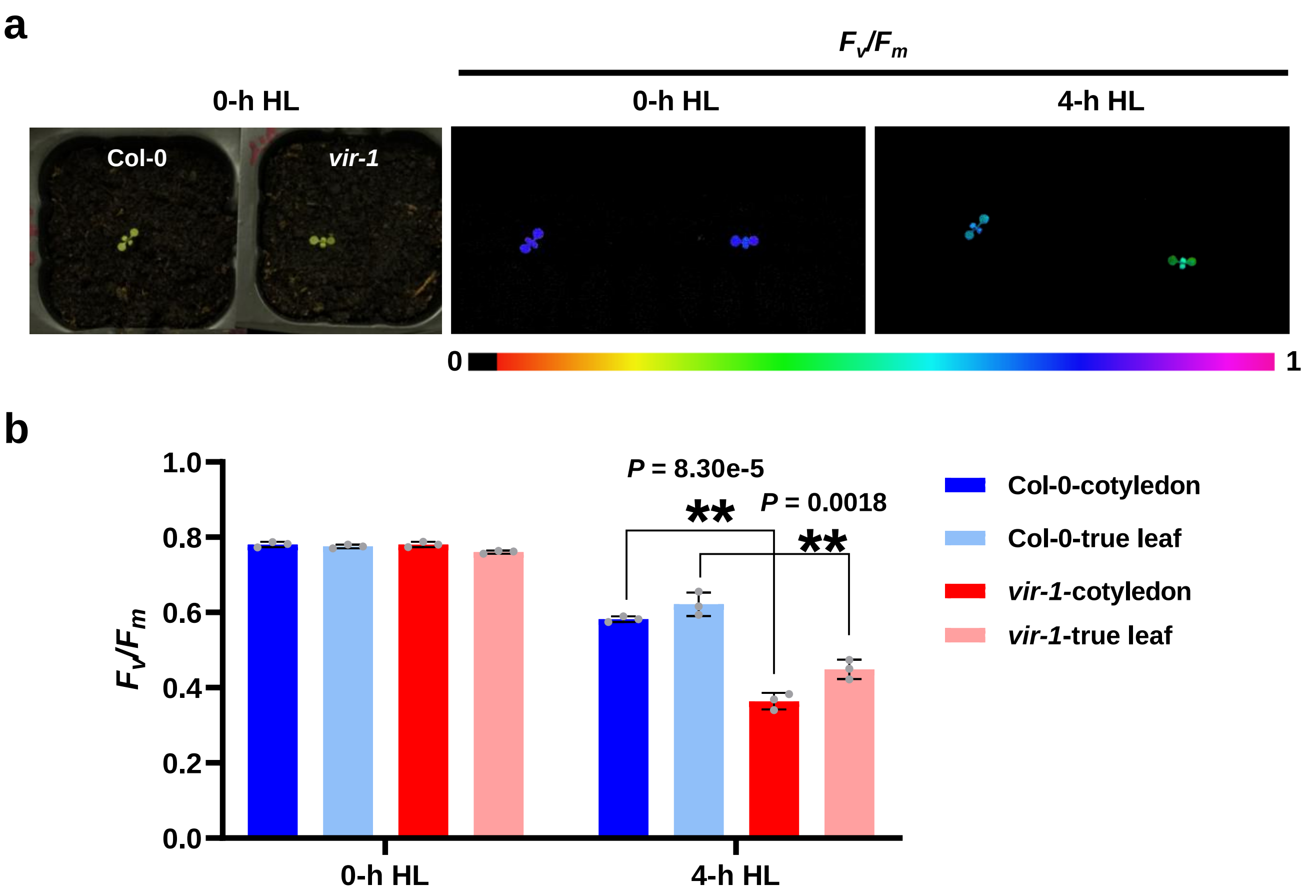


**Supplementary Figure 5. Analysis of photosynthetic activity in Col-0 and *vir-1* seedlings. a**, Left panel, image of Col-0 and *vir-1* seedlings with similar developmental stages under normal growth light grown on soil. Right panels, fluorescence images used to measure $F_v/F_m$ of plants grown under different light conditions. False-color images representing $F_v/F_m$ after a 0- and 4-h high light (HL) treatment in Col-0 and *vir-1* seedlings. The false color ranges from black (0) to purple (1), as indicated at the bottom. **b**, Changes in $F_v/F_m$ values for Col-0 and *vir-1* seedlings under HL treatment. Values are means ± SE (n = 3 biological replicates). *, $P < 0.05$; **, $P < 0.01$, by two-sided Student's *t*-test.

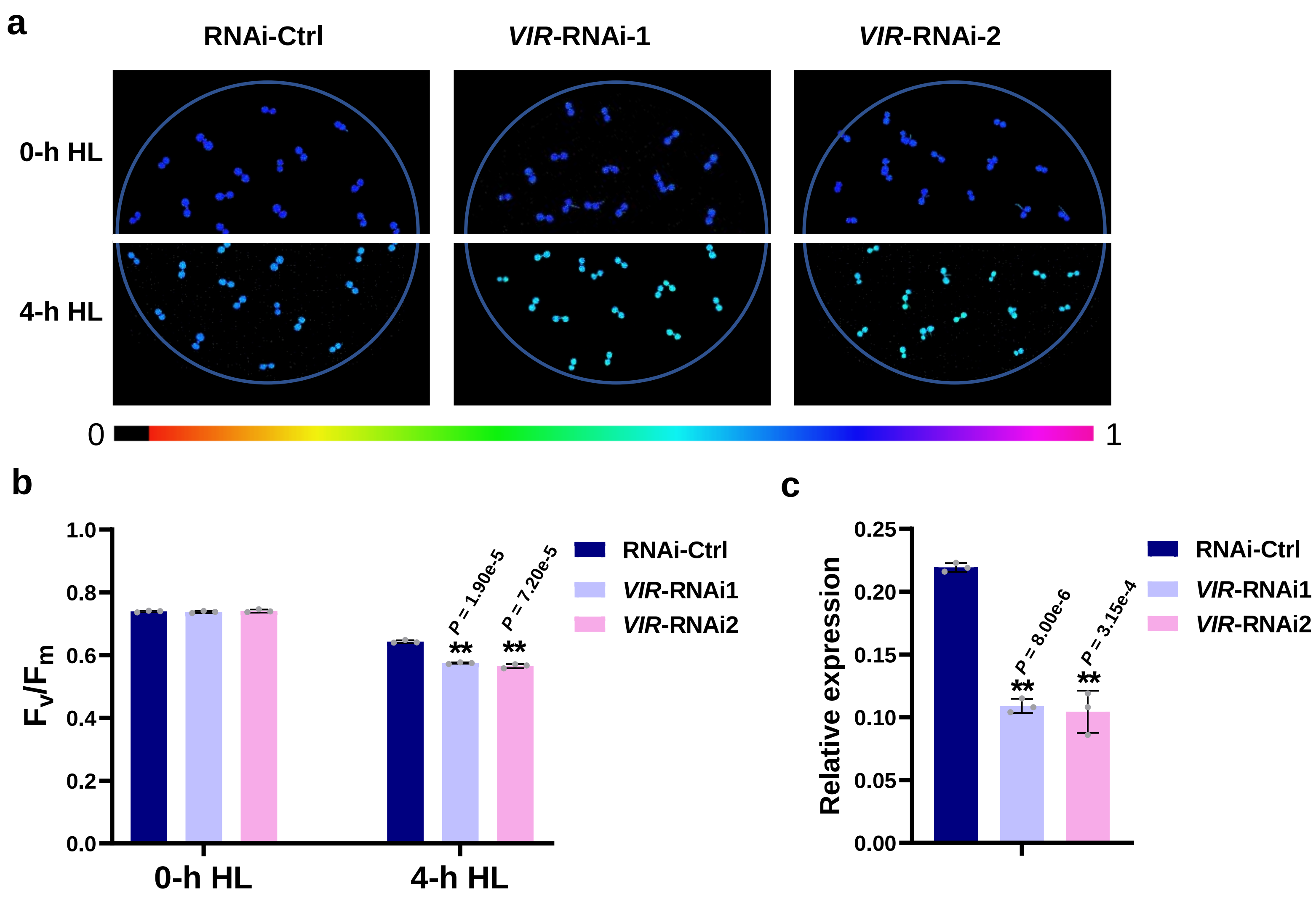


**Supplementary Figure 6. Analysis of photosynthetic activity in *VIR* RNAi seedlings. a,** Fluorescence images used to measure $F_v/F_m$ of seedlings grown under different light conditions. False-color images representing $F_v/F_m$ after a 0- and 4-h high light (HL) treatment in β-estradiol inducible RNAi-Ctrl, *VIR*-RNAi-1 and *VIR*-RNAi-2 seedlings are shown. The false color ranges from black (0) to purple (1), as indicated in the scale at the bottom. For the *VIR* RNAi line and the corresponding control, plant materials were first grown without β-estradiol for 4 days, then sprayed with 50 μM β-estradiol for 3 days and then subjected to 4 h of HL treatment. **b,** Changes in $F_v/F_m$ values for 7-day-old RNAi-Ctrl, *VIR*-RNAi-1 and *VIR*-RNAi-2 seedlings under HL treatment. Values are means ± SE (n = 3 biological replicates). *, $P < 0.05$; **, $P < 0.01$, by two-sided Student's *t*-test. **c**, Reduced expression of *VIR* was observed in inducible *VIR*-RNAi-1 and *VIR*-RNAi-2 seedlings. *ACTIN* was used as an internal control. Values are means ± SE (n = 3 biological replicates). *, $P < 0.05$; **, $P < 0.01$, by two-sided Student's *t*-test.

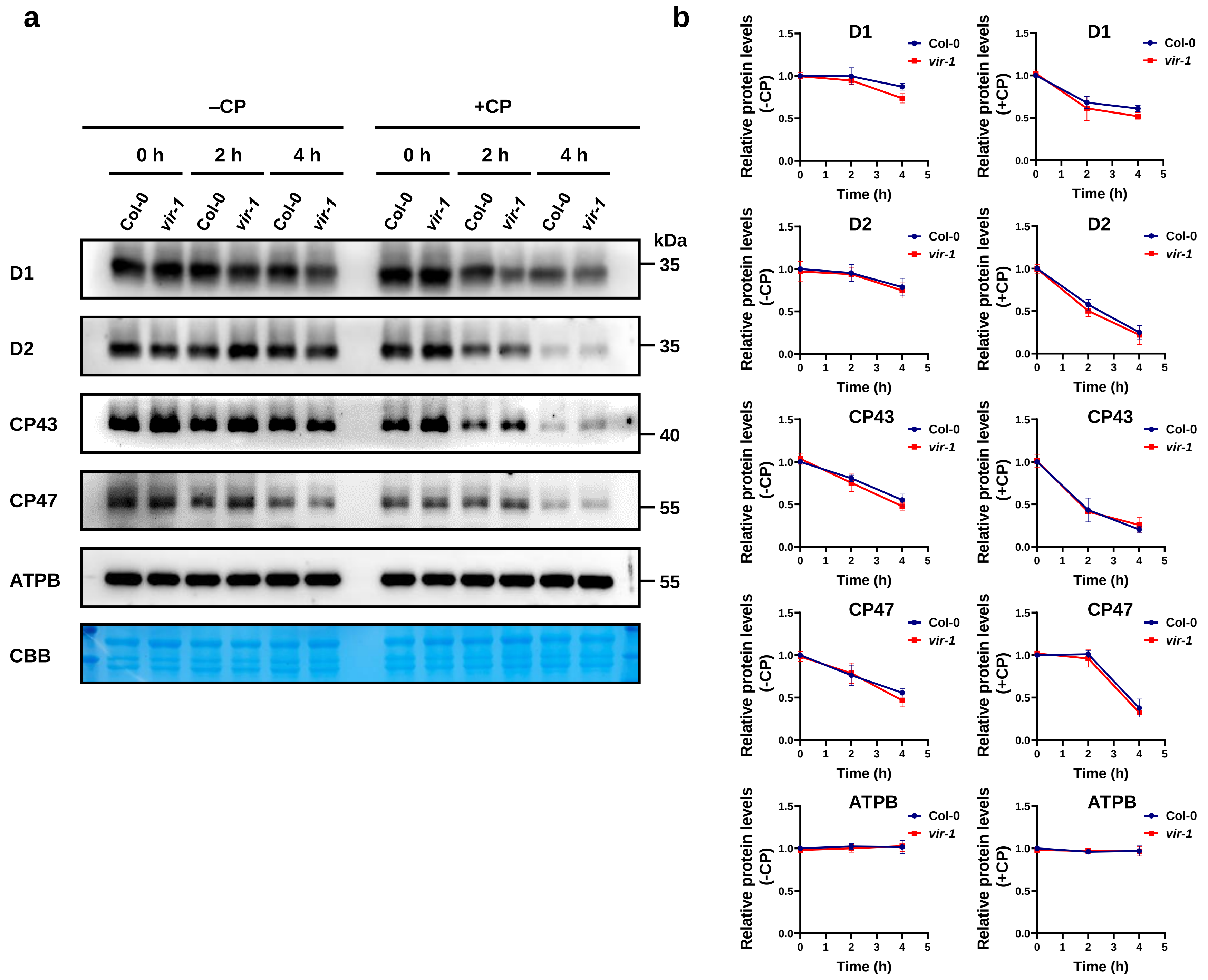


**Supplementary Figure 7. Degradation of PSII proteins in Col-0 and *vir-1* under high light treatment. a**, Thylakoid membrane proteins were isolated from Col-0 and *vir-1* plants during exposure to HL in the absence (–CP) or presence of 200 μg ml$^{-1}$ chloramphenicol (+CP), and PSII and ATPB protein levels were determined by immunoblot analysis. Lanes in each gel were loaded on an equal chlorophyll basis. CBB, Coomassie brilliant blue staining. All experiments were repeated three times with similar results. **b**, Proteins immunodetected from (**a**) were quantified with Phoretix 1D Software (Phoretix International, UK). Values (mean ± SE, *n* = 3 independent biological replicates) are given relative to protein levels of Col-0 before HL treatment. No significant differences were detected between Col-0 and *vir-1* by two-sided Student's *t*-test.

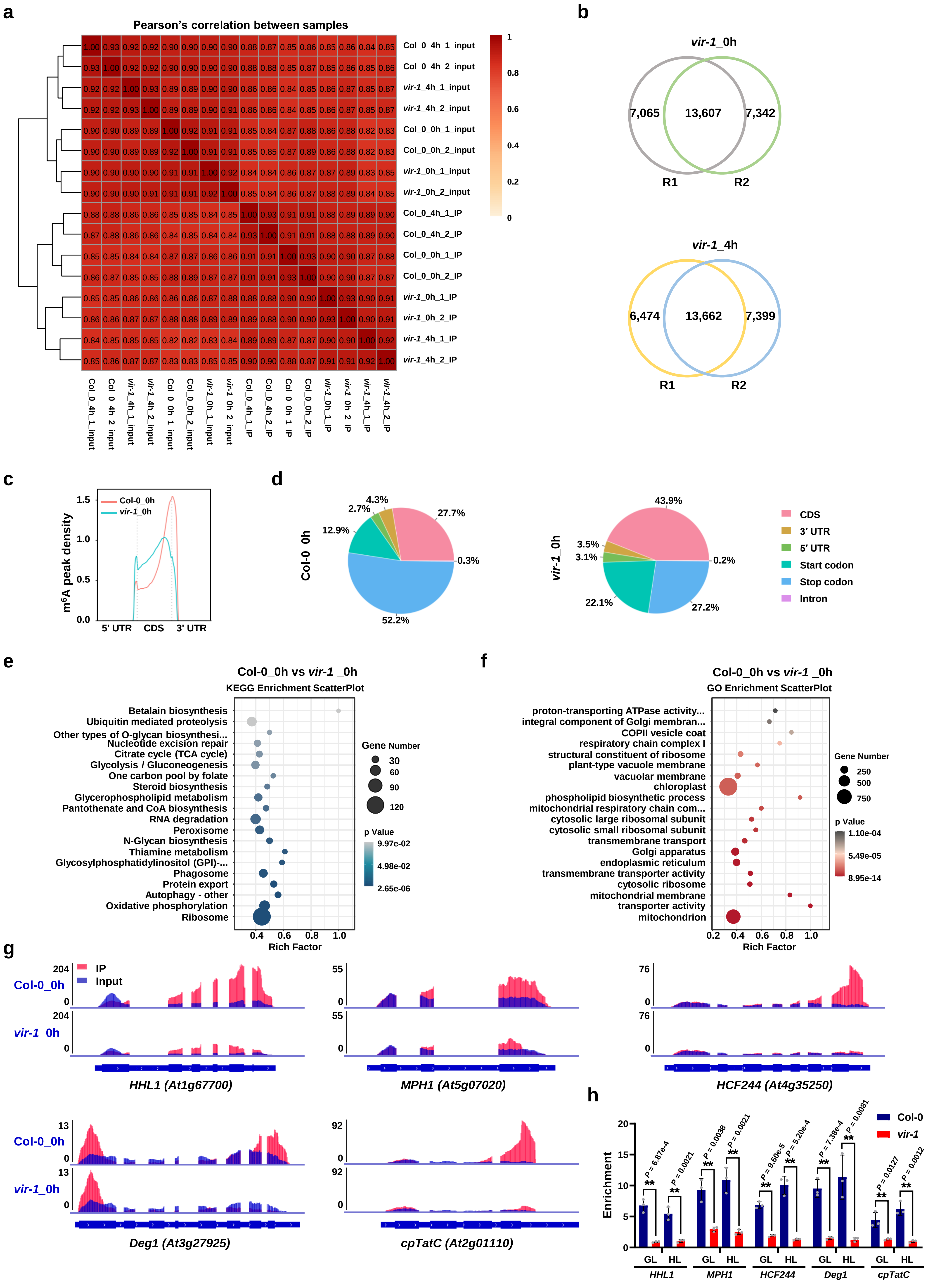

**Supplementary Figure 8. Comparation of m⁶A modifications between Col-0 and *vir-1*.** **a**, Pearson's correlation coefficients between the m⁶A profiles from two biological replicates of Col-0 and *vir-1* seedlings before (0 h) and after a 4-h high light (HL) treatment. **b**, Extent of overlap in m⁶A peaks identified in biological replicates (R1-R2) for *vir-1*_0h and *vir-1*_4h. **c**, Comparison of the distribution of m⁶A peaks along transcript segments in Col-0 and *vir-1* seedlings under normal growth light conditions. **d**, Comparison of the distribution of m⁶A peaks in different segments of Col-0 and *vir-1* transcripts under normal growth light conditions. CDS, coding sequence; UTR, untranslated region. **e**, KEGG analysis of VIR-dependent m⁶A -containing genes under normal growth light conditions. Statistical test was determined by one-sided hypergeometric test. **f**, GO analysis of VIR-dependent m⁶A -containing genes under normal growth light conditions. Statistical test was determined by one-sided hypergeometric test. **g**, Loss of VIR function results in lower (or loss of) m⁶A peaks compared to those in Col-0 seedlings under normal growth light conditions. Blue, input reads; red, IP reads. The gene models are shown below, with thick boxes and lines representing exons and introns, respectively. **h**, Validation of m⁶A sequencing results. Col-0 and *vir-1* seedlings before (0 h) and after a 4-h HL treatment were used for m⁶A -IP-qPCR. The enrichment of each gene was determined by m⁶A -IP-qPCR, first normalized by *TUB2*, and then the ratio of the abundance of the IP sample against the input sample was calculated. Values are means ± SE (n = 3 biological replicates). Asterisks indicate statistically significant differences in m⁶A enrichment levels between *vir-1* and Col-0 seedlings. *, *P* < 0.05; **, *P* <0.01, by two-sided Student's *t*-test.

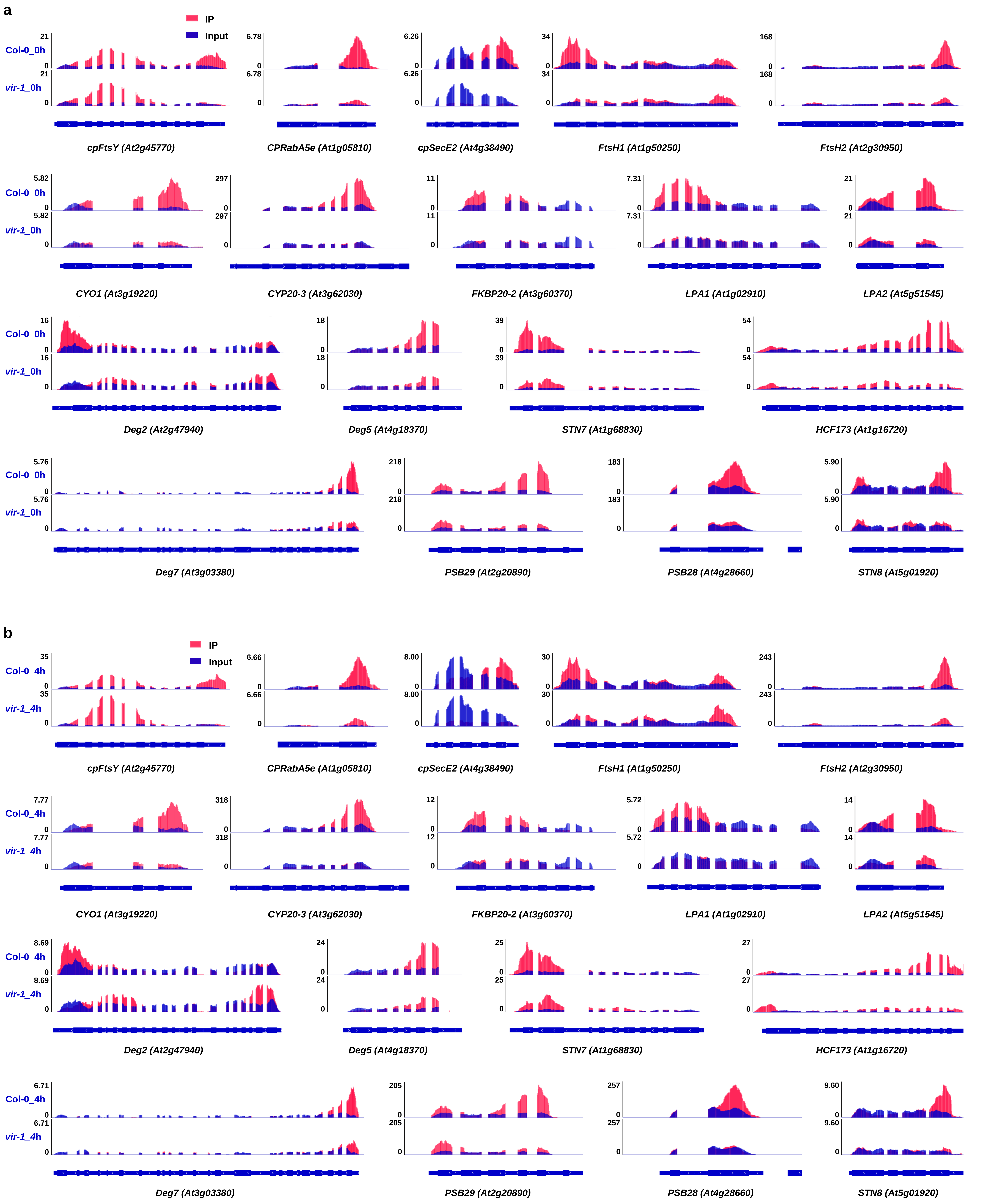


**Supplementary Figure 9. Analysis of m⁶A modifications of photoprotection-related mRNAs in *vir-1* mutants. a**, **b**, Loss of VIR function results in lower (or loss of) m⁶A peaks in photoprotection-related genes compared to those in Col-0 seedlings under normal growth light (**a**) or after a 4-h high light (HL) treatment (**b**). Blue, input reads; red, IP reads. The gene models are shown below, with thick boxes and lines representing exons and introns, respectively.

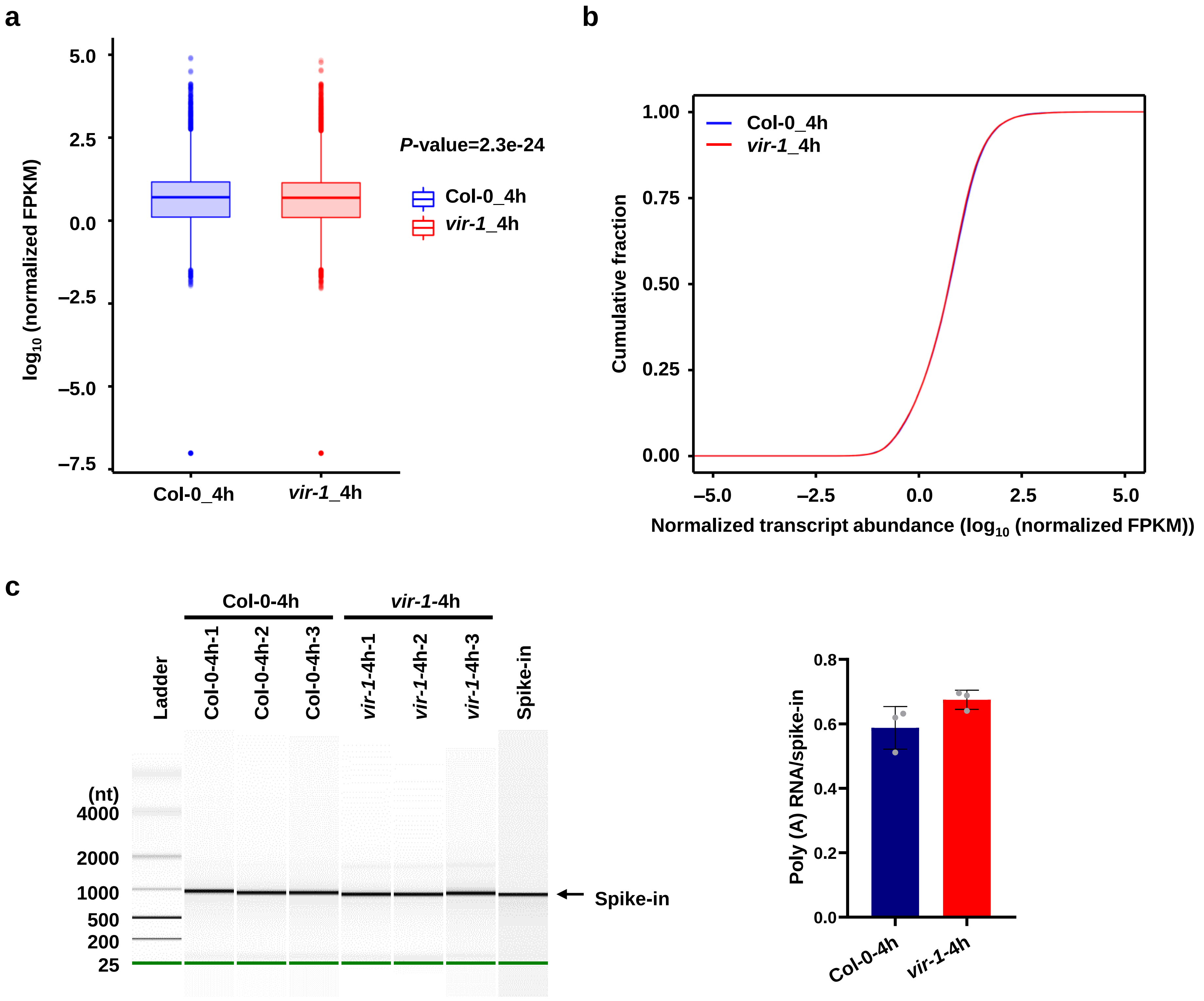


**Supplementary Figure 10. Quantification of poly(A) RNA in Col-0 and *vir-1*. a**, **b,** Box plots (**a**) and cumulative frequency plots (**b**) showing the distribution of transcript expression in 7-d-old Col-0 and *vir-1* seedlings after a 4-h HL treatment. In box plots, lower and upper hinges represent first and third quartiles, the center line represents the median, and whiskers represent ±1.5× the interquartile range. *P*-value were determined using two-sided Student's *t*-test. **c**, Ratio of poly(A) RNA to a synthesized poly(A) RNA spike-in control in a total RNA Pico Chip analysis using an Agilent 2100 Bioanalyzer. Poly(A) RNA along with the spike-in control were isolated from the same mass of 7-d-old Col-0 and *vir-1* seedlings after 4 h of HL treatment. Values are means ± SE (n = 3 biological replicates). No significant differences were detected between Col-0-4h and *vir-1*-4h by two-sided Student's *t*-test. nt, nucleotides.

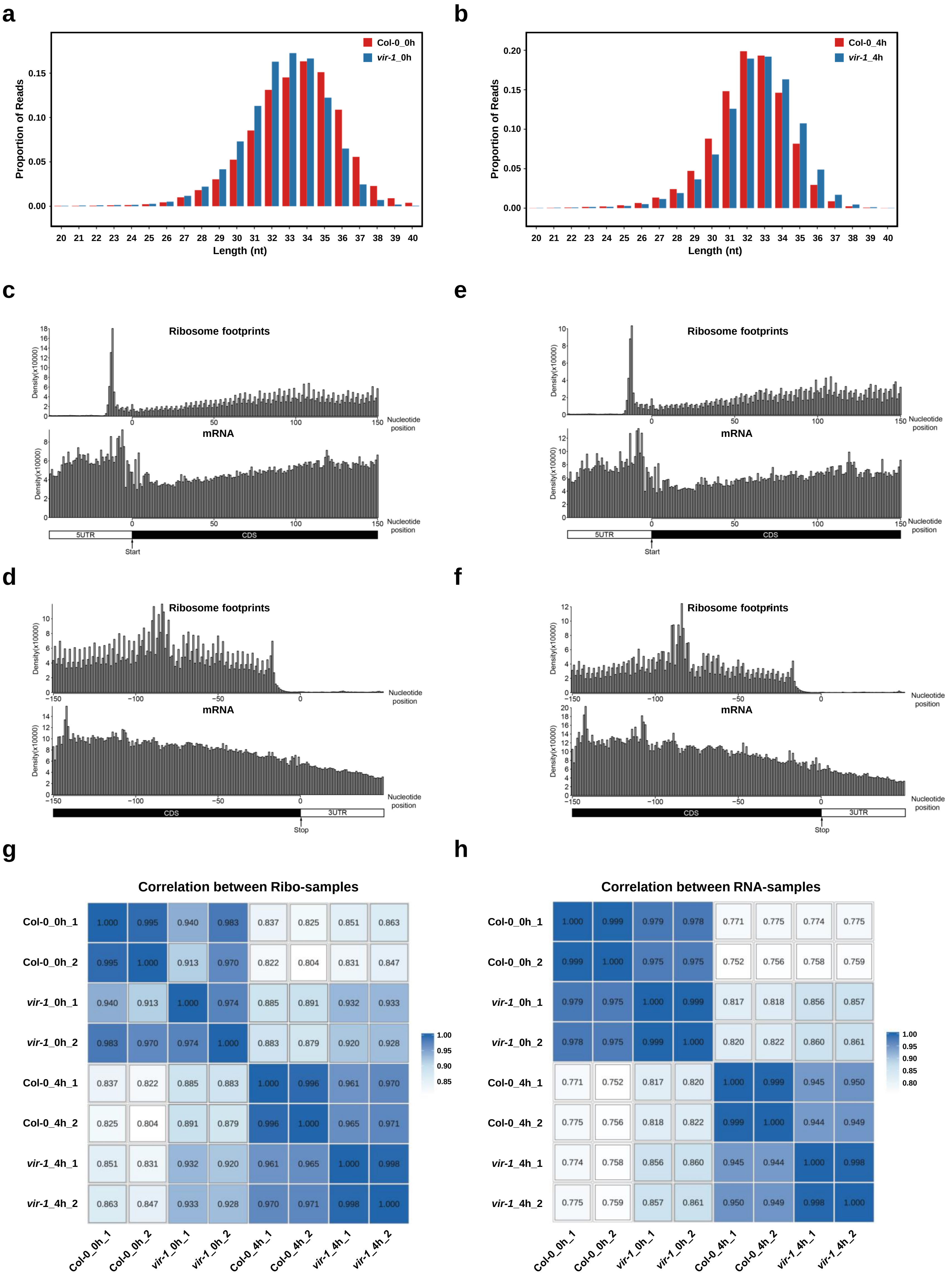


**Supplementary Figure 11. Quality and reproducibility of Ribo-seq and RNA-seq libraries. a**, **b**, Distribution of read length in the Col-0 and *vir-1* ribosome profiling data before (0 h, **a**) or after a 4-h high light (HL, **b**). **c**, **d**, Distribution of the ribosome footprints and mRNA reads for Col-0 near the start codons and stop codons of all coding sequences (CDSs). **e**, **f**, Distribution of the ribosome footprints and mRNA reads for *vir-1* near the start codons and stop codons of all CDSs. **g**, Pearson's correlation coefficients between ribosome profiling data from two biological replicates of Col-0 and *vir-1* before (0-h HL) and after 4-h HL treatment. **h**, Pearson's correlation coefficients between the gene expression profiles from two biological replicates of Col-0 and *vir-1* before (0-h HL) and after a 4-h HL treatment.

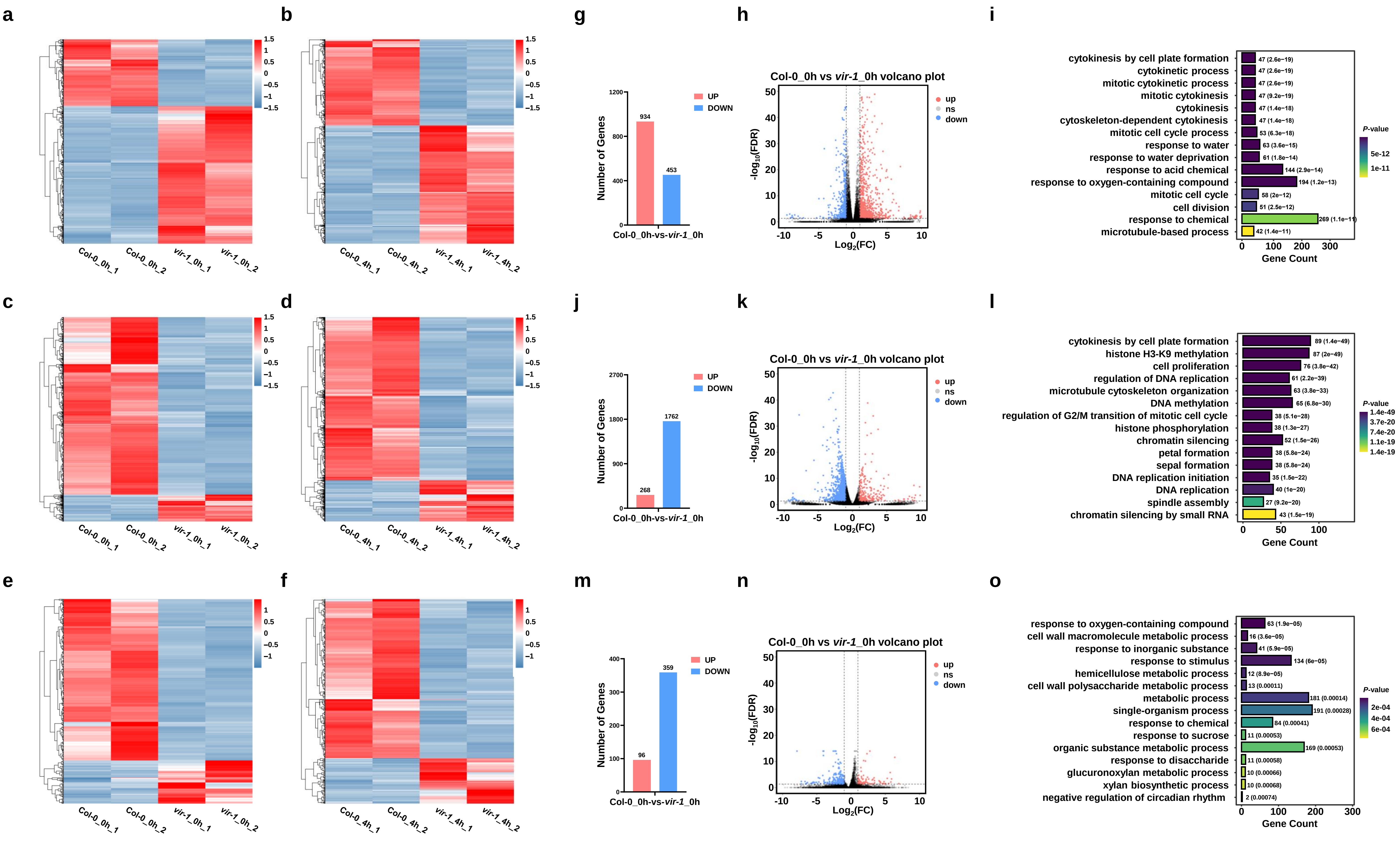


**Supplementary Figure 12. Transcriptome and translatome analysis of Col-0 and *vir-1*.** **a**, **b**, Heatmaps of the differentially expressed genes between Col-0 and *vir-1* before (**a**) and after (**b**) HL treatment. Red, higher expression levels; blue, lower expression levels. The values displayed in the heatmaps (a, b) are FPKM values from all conditions. The units shown in the heatmap (a, b) were Z-score normalized. **c**, **d**, Heatmaps of the differentially translated genes between Col-0 and *vir-1* before (**c**) and after (**d**) HL treatment. Red, higher translation levels; blue, lower translation levels. The values displayed in the heatmaps (c,d) are FPKM values from all conditions. The units shown in the heatmap (c,d) were Z-score normalized. **e**, **f**, Heatmaps of the genes showing differential TE between Col-0 and *vir-1* before (**e**) and after (**f**) HL treatment. Red, higher TE levels; blue, lower TE levels. The values displayed in the heatmaps (e, f) are FPKM values from all conditions. The units shown in the heatmap (e, f) were Z-score normalized. **g**, Number of differentially expressed genes between Col-0 and *vir-1* under growth light conditions. **h**, Volcano plot of differentially expressed genes between Col-0 and *vir-1* under normal growth light conditions. Significantly downregulated genes are shown in blue, significantly upregulated genes are shown in red, and genes without significant differences in expression are shown in black. Black vertical lines highlight $Log_2$(fold-change) = 1 or –1; black horizontal line represents an FDR of 0.05. **i**, GO enrichment analysis of the differentially expressed genes between Col-0 versus *vir-1* under growth light conditions. Statistical test was determined by one-sided hypergeometric test. **j**, Number of differentially translated genes between Col-0 and *vir-1* under growth light conditions. **k**, Volcano plot of differentially translated genes between Col-0 and *vir-1* under growth light conditions, indicated as in (h). **l**, GO enrichment analysis of the differentially translated genes between Col-0 and *vir-1* under growth light conditions. Statistical test was determined by one-sided hypergeometric test. **m**, Number of genes showing differential TE between Col-0 and *vir-1* under growth light conditions. **n**, Volcano plot of the genes showing differential TE between Col-0 and *vir-1* under growth light conditions, indicated as in (h). **o**, GO enrichment analysis of the genes showing differential TE between Col-0 and *vir-1* under growth light conditions. Statistical test was determined by one-sided hypergeometric test.

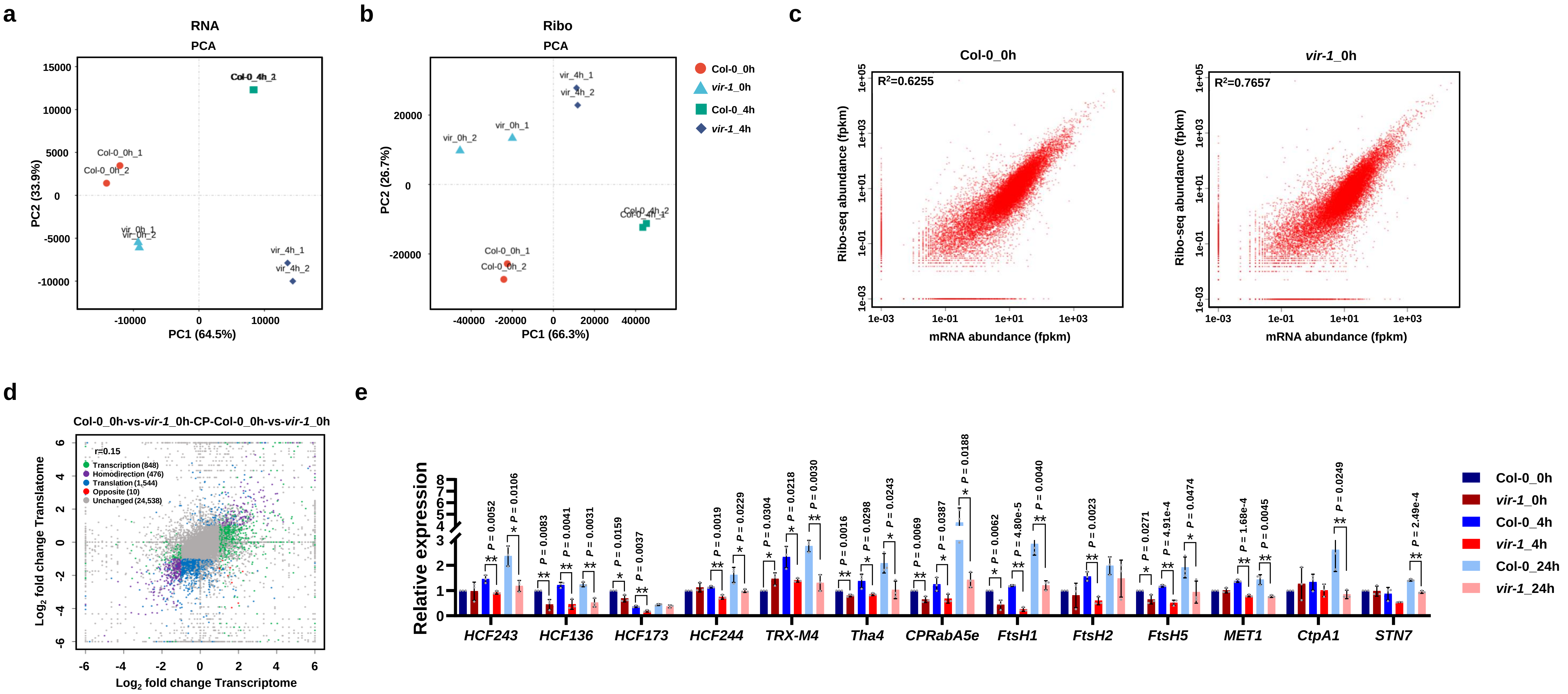


**Supplementary Figure 13. Principal component analysis and correlation analysis of RNA-seq samples and Ribo-seq samples. a**, Principal component analysis of RNA-seq samples. **b**, Principal component analysis of Ribo-seq samples. **c**, Extent of correlation between RNA-seq and Ribo-seq samples under normal growth light conditions. Pearson's correlation coefficient (r) is shown. **d**, Extent of correlation between the transcriptome changes and translatome changes in *vir-1 vs.* Col-0 under 0-h high light treatment. Pearson's correlation coefficient (*r*) is shown. **e**, Relative expression levels of photoprotection-related genes in Col-0 and *vir-1* under high light conditions. *UBQ10* was used as an internal control. Values are means ± SE (n = 3 biological replicates). *, *P* < 0.05; **, *P* < 0.01, by two-sided Student's *t*-test.

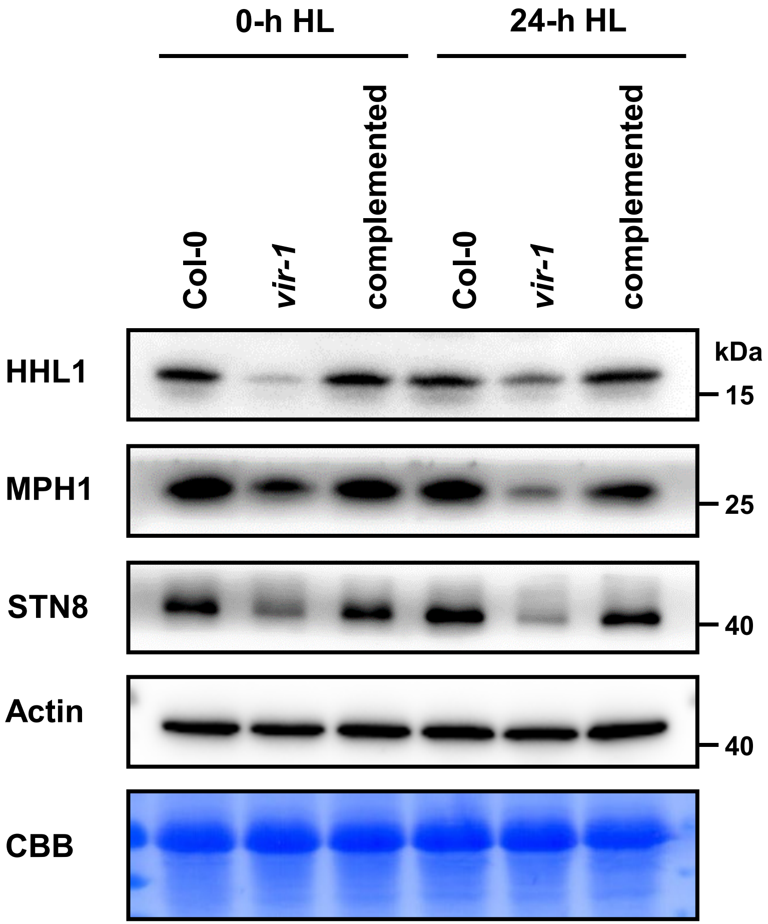


**Supplementary Figure 14. Analysis of protein abundance in Col-0, *vir-1*, and complemented seedlings.** Protein samples from Col-0, *vir-1*, and complemented seedlings were separated by 12% SDS-urea-PAGE and probed with antisera against specific proteins. CBB was used to estimate loading. Similar results were obtained from three independent biological replicates. HL, high light.

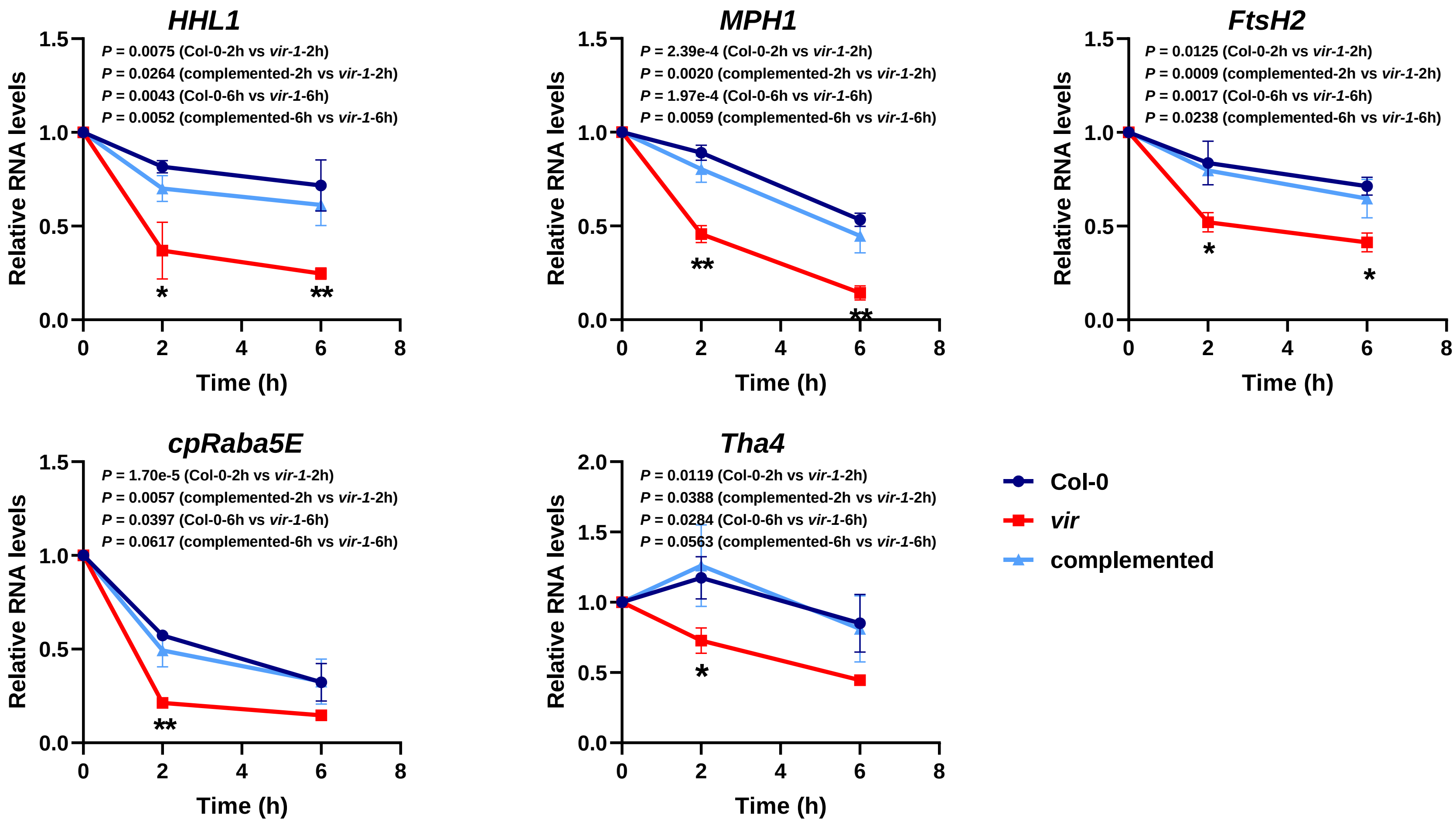


**Supplementary Figure 15. mRNA lifetimes of *HHL1*, *MPH1*, *FtsH2*, *cpRaba5E*, and *Tha4* in Col-0, *vir-1*, and complemented seedlings.** Seven-day-old Col-0, *vir-1*, and complemented seedlings treated with actinomycin D for 0, 2, or 6 h were used for transcription inhibition assays. 18S ribosomal RNA was used as the internal reference. Values are means ± SE (n = 3 biological replicates). *, $P < 0.05$; **, $P < 0.01$, by two-sided Student's *t*-test.

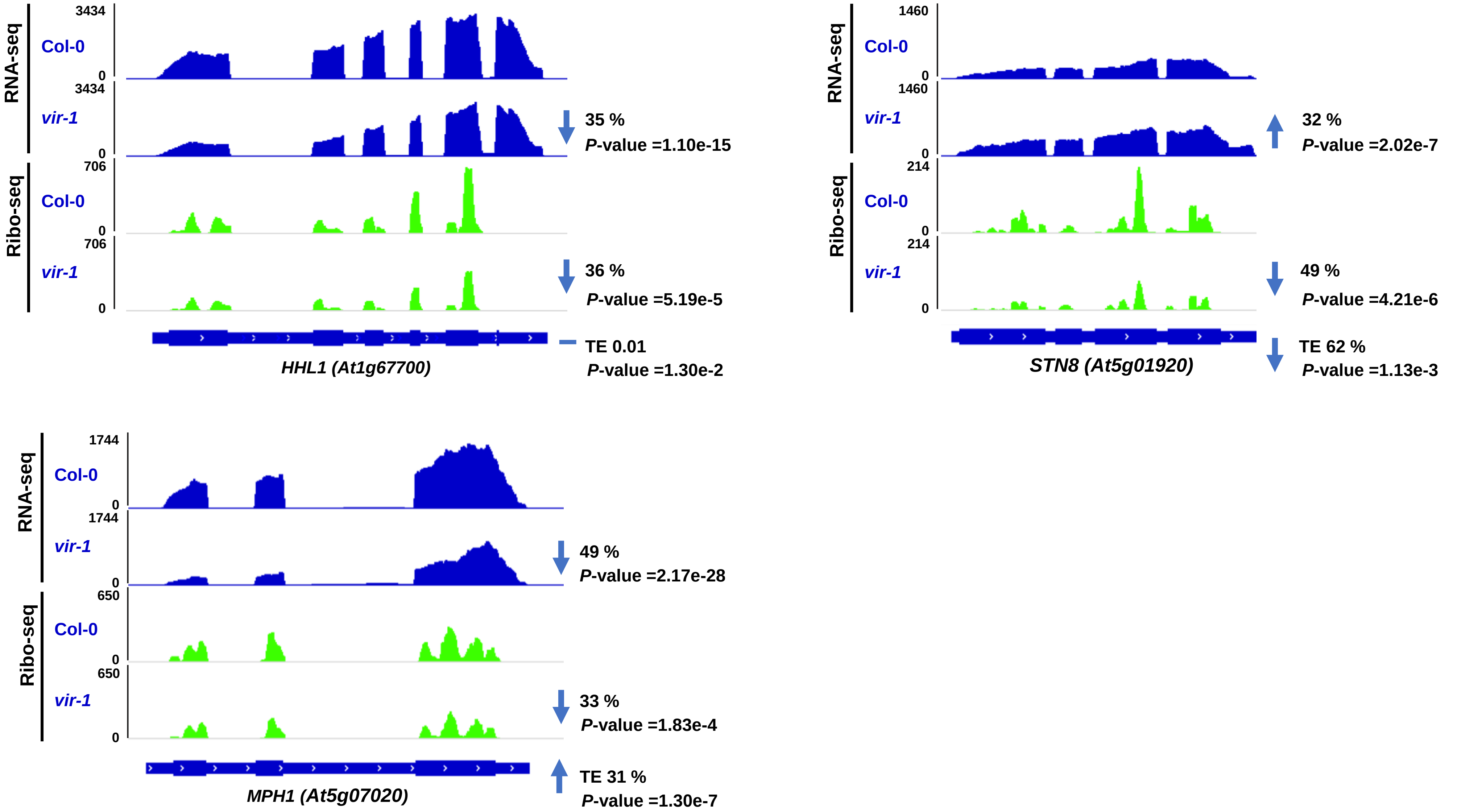


**Supplementary Figure 16. Normalized distribution of RNA-seq and Ribo-seq reads in Col-0 and *vir-1* along the *HHL1*, *STN8*, and *MPH1* genes before high light treatment.** The fold-change and the associated *P*-value for VIR effect on transcript and footprint levels, as well as the fold-change in the footprint levels given the levels of mRNA (TE) and the corresponding *P*-value, are shown. The *P*-value of RNA-seq and Ribo-seq was calculated by negative binomial distribution model. The *P*-value of TE was calculated by RiboDiff.

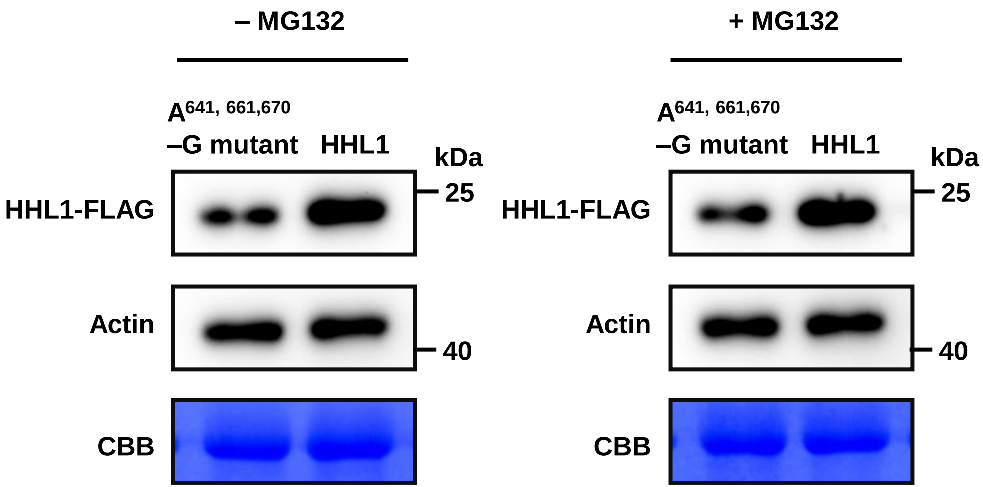


**Supplementary Figure 17. Analysis of HHL1 protein stability in *hhl1* protoplasts transfected with equal amounts of wild-type or mutant *HHL1* plasmids.** $A^{641,\ 661,\ 670}$-G mutant, mutant harboring the transition mutations A641, A661, A670-G. The overnight cultured protoplasts were treated with 50 μM MG132 (+ MG132) or without MG132 (–MG132). Similar results were obtained from three independent biological replicates.

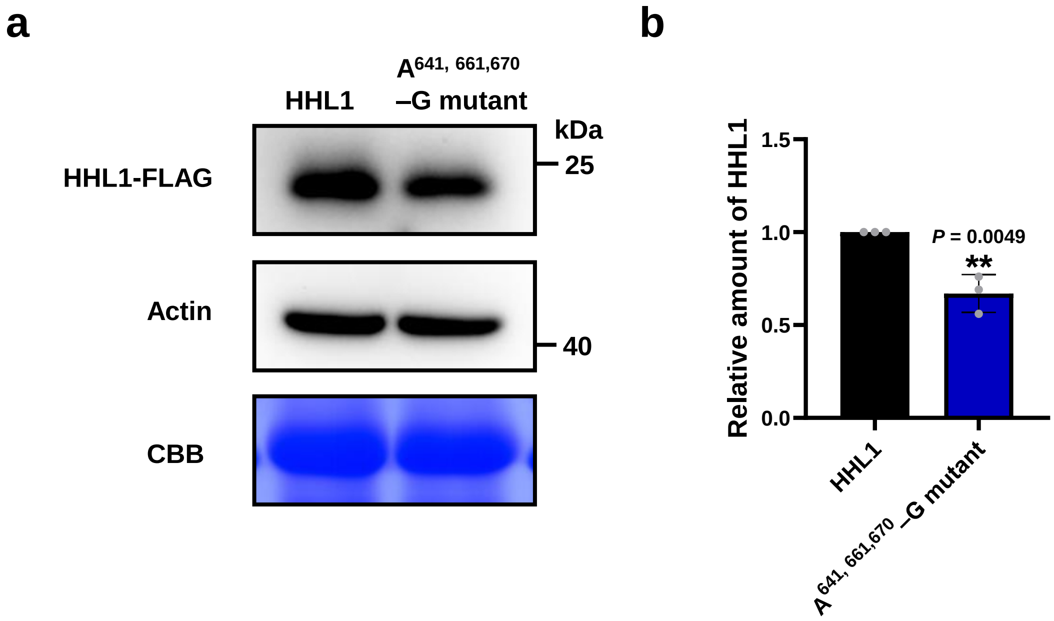


**Supplementary Figure 18. Analysis of wild-type or mutant HHL1 accumulation in *vir-1* protoplasts. a**, HHL1-FLAG accumulation in *vir-1* protoplasts transfected with equal amounts of plasmids overexpressing wild-type or mutant *HHL1*. $A^{641,\ 661,\ 670}$-G mutant, mutant harboring the transition mutations A641, A661, A670-G. **b,** Proteins immunodetected from (**a**) were quantified with Phoretix 1D Software (Phoretix International, UK). Values (means $\pm$ SE; $n$ = 3 independent biological replicates) are given relative to protein levels of wild-type HHL1. *, $P < 0.05$; **, $P < 0.01$, by two-sided Student's $t$-test.

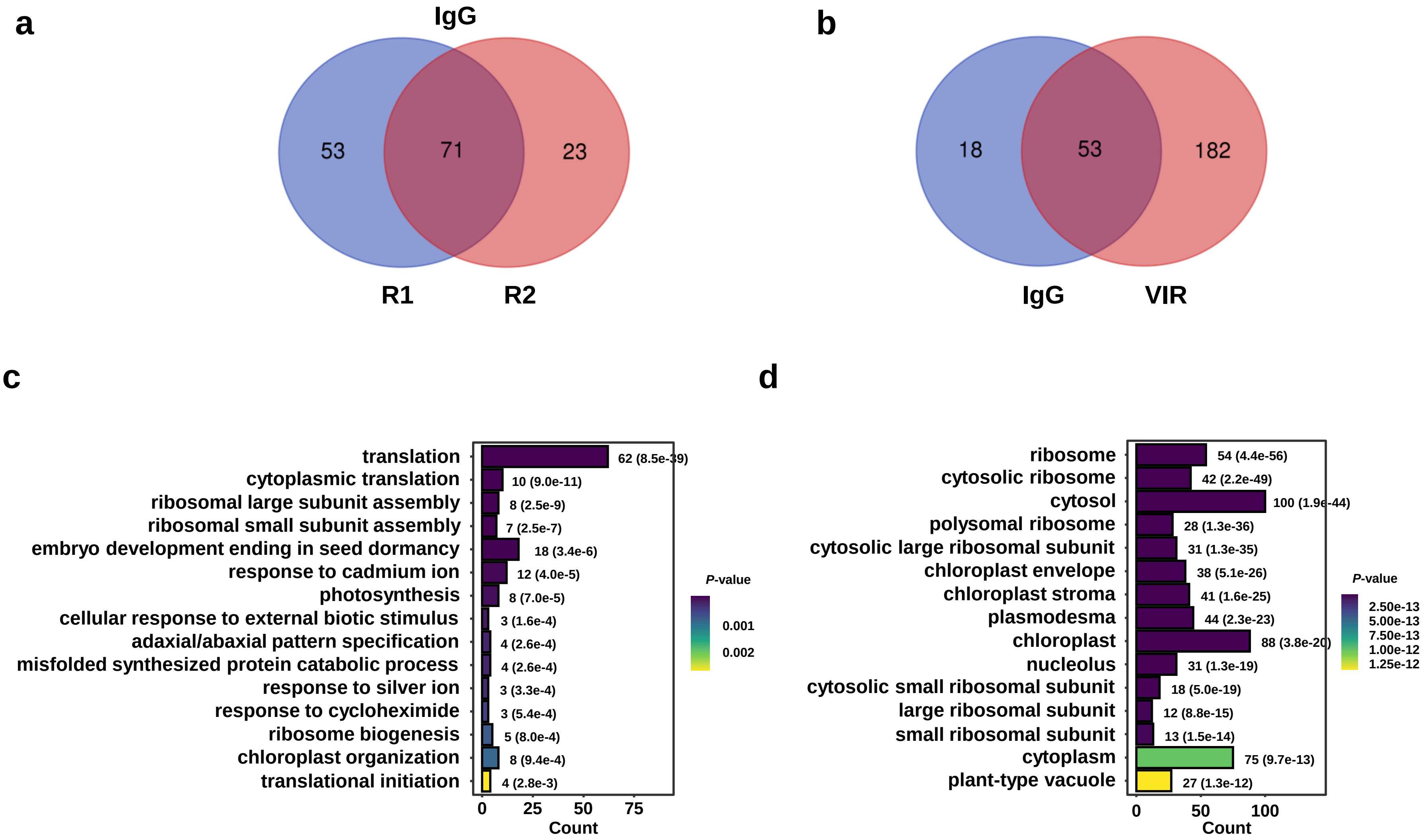


**Supplementary Figure 19. LC-MS/MS related to Figure 7. a**, Overlap in proteins identified in biological replicates (R1-R2) of control IgG samples. **b**, Venn diagram showing the extent of overlap in proteins between IgG and VIR samples. **c**, **d**, GO enrichment analysis of biological processes (**c**) and cellular components (**d**) of VIR-associated proteins. Statistical test was determined by one-sided hypergeometric test.

**Supplementary Table 1. Chlorophyll fluorescence parameters in the wild-type (Col-0), *vir-1* mutants and the complemented lines.**

| | Growth Light | | | After 4-h High Light | | | After 4-h HL and 2-day recovery | | |
|---|---|---|---|---|---|---|---|---|---|
| | Col-0 | *vir-1* | complement | Col-0 | *vir-1* | complement | Col-0 | *vir-1* | complement |
| Fm' | 0.590±0.016 | 0.566±0.003 | 0.570±0.019 | 0.254±0.023 | 0.218±0.005 | 0.227±0.014 | 0.587±0.014 | 0.578±0.013 | 0.604±0.005 |
| Fo' | 0.130±0.003 | 0.131±0.001 | 0.131±0.004 | 0.109±0.011 | 0.131±0.004 | 0.095±0.007 | 0.117±0.003 | 0.121±0.003 | 0.127±0.003 |
| Y(II) | 0.780±0.001 | 0.768±0.004 | 0.770±0.001 | 0.571±0.010 | 0.398±0.008$^{**}$ | 0.580±0.018 | 0.800±0.001 | 0.790±0.002 | 0.789±0.002 |
| qP | 0.697±0.021 | 0.524±0.001$^{**}$ | 0.693±0.013 | 0.825±0.011 | 0.804±0.040 | 0.777±0.019 | 0.720±0.006 | 0.594±0.012$^{**}$ | 0.703±0.007 |
| qL | 0.468±0.019 | 0.292±0.002$^{**}$ | 0.472±0.016 | 0.670±0.023 | 0.721±0.047 | 0.590±0.035 | 0.463±0.006 | 0.325±0.008$^{**}$ | 0.465±0.004 |

**Supplementary Table 2. Multi-omics sequencing read counts and mapping rates.**

**$m^6A$-seq sequencing read counts and mapping rates**

| Sample | | Total Reads | Unique Mapped Reads | Multiple Mapped reads | Mapping Ratio |
|---|---|---|---|---|---|
| Col-0_0h_IP | Rep 1 | 52411540 | 37270018(71.11%) | 14333102(27.35%) | 51603120(98.46%) |
| | Rep 2 | 52238472 | 37792558(72.35%) | 13573192(25.98%) | 51365750(98.33%) |
| *vir-1*_0h_IP | Rep 1 | 53864502 | 35339515(65.61%) | 17778140(33.01%) | 53117655(98.61%) |
| | Rep 2 | 50820896 | 32931132(64.80%) | 17227086(33.90%) | 50158218(98.70%) |
| Col-0_4h_IP | Rep 1 | 51529112 | 38860172(75.41%) | 11864194(23.02%) | 50724366(98.44%) |
| | Rep 2 | 53923758 | 41715307(77.36%) | 11505138(21.34%) | 53220445(98.70%) |
| *vir-1*_4h_IP | Rep 1 | 51121082 | 36427755(71.26%) | 13859833(27.11%) | 50287588(98.37%) |
| | Rep 2 | 52676272 | 38361714(72.83%) | 13701921(26.01%) | 52063635(98.84%) |
| Col-0_0h_input | Rep 1 | 51917712 | 28097250(54.12%) | 22558633(43.45%) | 50655883(97.57%) |
| | Rep 2 | 53496820 | 28524141(53.32%) | 24161458(45.16%) | 52685599(98.48%) |
| *vir-1*_0h_input | Rep 1 | 50714020 | 27967391(55.15%) | 21950157(43.28%) | 49917548(98.43%) |
| | Rep 2 | 51449080 | 28800413(55.98%) | 21984974(42.73%) | 50785387(98.71%) |
| Col-0_4h_input | Rep 1 | 55531476 | 30500058(54.92%) | 24159804(43.51%) | 54659862(98.43%) |
| | Rep 2 | 54802378 | 31743320(57.92%) | 22133832(40.39%) | 53877152(98.31%) |
| *vir-1*_4h_input | Rep 1 | 53237992 | 28777521(54.05%) | 23568533(44.27%) | 52346054(98.32%) |
| | Rep 2 | 52566944 | 29839388(56.76%) | 22022201(41.89%) | 51861589(98.66%) |

**RNA-seq sequencing read counts and mapping rates**

| Sample | | Total Reads | Unique Mapped Reads | Multiple Mapped reads | Mapping Ratio |
|---|---|---|---|---|---|
| Col-0_0h | Rep 1 | 38471736 | 36912869 (95.95%) | 791243 (2.06%) | 37704112 (98.00%) |
| | Rep 2 | 38848838 | 37324226 (96.08%) | 786203 (2.02%) | 38110429 (98.10%) |
| *vir-1*_0h | Rep 1 | 83569664 | 79952879 (95.67%) | 1884599 (2.26%) | 81837478 (97.93%) |
| | Rep 2 | 40015926 | 37753863 (94.35%) | 879935 (2.20%) | 38633798 (96.55%) |

| | | | | | |
|---|---|---|---|---|---|
| Col-0_4h | Rep 1 | 38937558 | 37394734 (96.04%) | 881318 (2.26%) | 38276052 (98.30%) |
| | Rep 2 | 39460072 | 37787497 (95.76%) | 894257 (2.27%) | 38681754 (98.03%) |
| *vir-1*_4h | Rep 1 | 37564548 | 35928615 (95.65%) | 926273 (2.47%) | 36854888 (98.11%) |
| | Rep 2 | 57249618 | 54879051 (95.86%) | 1480513 (2.59%) | 56359564 (98.45%) |

**Ribo-seq Sequencing read counts and mapping rates**

| Sample | | Total Reads | Unique Mapped Reads | Multiple Mapped reads | Mapping Ratio |
|---|---|---|---|---|---|
| Col-0_0h | Rep 1 | 20995321 | 12985720 (61.85%) | 3710068 (17.67%) | 16695788 (79.52%) |
| | Rep 2 | 19219672 | 11931599 (62.08%) | 3389088 (17.63%) | 15320687 (79.71%) |
| *vir-1*_0h | Rep 1 | 23232523 | 15057799 (64.81%) | 4404296 (18.96%) | 19462095 (83.77%) |
| | Rep 2 | 20960296 | 12928809 (61.68%) | 3920194 (18.70%) | 16849003 (80.39%) |
| Col-0_4h | Rep 1 | 23726831 | 17228728 (72.61%) | 3679031 (15.51%) | 20907759 (88.12%) |
| | Rep 2 | 24439767 | 17922265 (73.33%) | 3551259 (14.53%) | 21473524 (87.86%) |
| *vir-1*_4h | Rep 1 | 16341448 | 11033898 (67.52%) | 3236874 (19.81%) | 14270772 (87.33%) |
| | Rep 2 | 17104590 | 11400025 (66.65%) | 3312687 (19.37%) | 14712712 (86.02%) |

**Supplementary Table 3. Changes in $m^6A$ modification levels of chloroplast/photosynthesis-related genes after 4 h of high light treatment.**

| Gene | $log_2fc$ | function |
|---|---|---|
| AT3G25110 | 1.07 | UP |
| AT1G03730 | 1.10 | UP |
| AT3G18680 | 2.17 | UP |
| AT2G46220 | 1.15 | UP |
| AT5G15700 | 1.01 | UP |
| AT5G47435 | 1.77 | UP |
| AT2G07715 | 1.11 | UP |
| AT3G27550 | 1.13 | UP |
| AT5G41220 | 1.10 | UP |
| AT1G67120 | 1.05 | UP |
| AT1G06870 | 1.11 | UP |
| AT3G07520 | 1.20 | UP |
| AT4G25030 | -1.16 | DOWN |
| AT1G48490 | -1.02 | DOWN |
| AT5G53920 | -1.27 | DOWN |
| AT5G25140 | -1.09 | DOWN |
| AT4G04960 | -2.06 | DOWN |
| AT1G79460 | -1 | DOWN |
| AT3G14790 | -1.31 | DOWN |
| AT1G67930 | -1.29 | DOWN |
| AT3G07080 | -1.05 | DOWN |
| AT3G23020 | -1 | DOWN |
| AT1G48320 | -1.26 | DOWN |
| AT3G25130 | -1.10 | DOWN |
| AT1G49980 | -1.03 | DOWN |
| AT3G54660 | -1.05 | DOWN |
| AT1G01510 | -1.01 | DOWN |
| AT1G36180 | -1.51 | DOWN |
| AT1G14230 | -1.11 | DOWN |
| AT3G47680 | -1.05 | DOWN |
| AT1G56260 | -1.05 | DOWN |
| AT4G19510 | -1.02 | DOWN |
| AT5G08740 | -1.05 | DOWN |
| AT3G06380 | -1.26 | DOWN |
| AT1G09830 | -1 | DOWN |
| AT5G04110 | -1.27 | DOWN |
| AT1G55320 | -1.56 | DOWN |
| AT1G70070 | -1.06 | DOWN |
| AT5G57850 | -1.04 | DOWN |
| AT5G33280 | -1.04 | DOWN |

| | | |
|---|---|---|
| AT1G07780 | -1.41 | DOWN |
| AT2G43950 | -1.16 | DOWN |
| AT5G53460 | -1.03 | DOWN |
| AT3G23710 | -1.17 | DOWN |
| AT1G73820 | -1.04 | DOWN |
| AT1G76900 | -1.29 | DOWN |
| AT5G42310 | -1.13 | DOWN |
| AT5G20110 | -1.09 | DOWN |
| AT4G29530 | -1.42 | DOWN |
| AT4G27630 | -1.20 | DOWN |
| AT1G28150 | -1.12 | DOWN |
| AT1G63900 | -1.26 | DOWN |
| AT4G14740 | -1.25 | DOWN |
| AT2G26170 | -1.07 | DOWN |
| AT1G51760 | -1.20 | DOWN |
| AT5G41740 | -1.37 | DOWN |
| AT1G74890 | -1.60 | DOWN |
| AT3G15390 | -1.07 | DOWN |
| AT3G22150 | -1.27 | DOWN |
| AT2G29630 | -1.23 | DOWN |
| AT1G68730 | -1.06 | DOWN |
| AT2G27740 | -1.24 | DOWN |
| AT2G28880 | -1 | DOWN |
| AT5G06290 | -1.21 | DOWN |
| AT3G11330 | -1.67 | DOWN |
| AT5G35840 | -1.11 | DOWN |
| AT5G65430 | -1.35 | DOWN |
| AT5G22770 | -1.11 | DOWN |
| AT1G09390 | -1.50 | DOWN |
| AT2G47750 | -1.04 | DOWN |
| AT2G02950 | -1.06 | DOWN |
| AT2G36890 | -2.18 | DOWN |
| AT1G76550 | -1.03 | DOWN |
| AT3G20270 | -1.21 | DOWN |
| AT3G17790 | -1.22 | DOWN |
| AT5G64610 | -1.53 | DOWN |

**Supplementary Table 4. List of primers used in this study.**

| Primer | Sequence |
|---|---|
| $m^6A$ sequencing results verification | |
| AT1G06760-m6A-F | GGCTAAAGCATCATCCCCTA |
| AT1G06760-m6A-R | GCAGTCTTAGGTTTAACGGC |
| AT2G05070-m6A-F | GCCTGGTCTTACGCTACTAA |
| AT2G05070-m6A-R | ACGTCAATATTACACAATCACGA |
| AT3G60300-m6A-F | ATCCAAGAAAACCGACGAGA |
| AT3G60300-m6A-R | TCAGAAACAAATATGCTCACAGA |
| AT1G48850-m6A-F | CAGGAACCTCTCCAGATAG |
| AT1G48850-m6A-R | AAGCGAACTCTCACTCTC |
| HHL1-m6A-F | CGACAAAGTTAAAGACTACT |
| HHL1-m6A-R | TTTCTCATCCGAGGTTTC |
| HCF244-m6A-F | CCGATGACTGAGACTAATAG |
| HCF244-m6A-R | ATGTTGCTGAAGTAATCCT |
| Deg1-m6A-F | CTAAGAGGCGATCACAAG |
| Deg1-m6A-R | TTCAACGACGTTTCTAAGA |
| cpTatC-m6A-F | GTTCCAGTCATTCAGTTAC |
| cpTatC-m6A-R | CCTACCACCACATATCTC |
| MPH1-m6A-F | ACATCAACAAGTTCAAGCCTTC |
| MPH1-m6A-R | GAGTTTCTGGCAGTGAAGTTTC |
| Mutant identification | |
| VIR-JC-F | AGCGATTGTTGCAGCTGCTG |
| VIR-JC-R | AGAGTGGCTTTCCCCTGAAATG |
| Alternative splicing | |
| VIR_E4_F | TTCTTGAATCAGAGACTTATTTGGC |
| VIR_E6_R | TTAGAGAGACCTTCCAGTGC |
| Quantitative real-time PCR | |
| VIR-qPCR-F | ACGCAAGTCCAGCCTTACTATCAC |
| VIR-qPCR-R | CGGTCACTTAATAGAGCCTGAATGG |
| MTB-qPCR-F | GTACTGTGTTTCAGCGTTCC |
| MTB-qPCR-R | TTCTGAGTCGAACCATAAGGAG |
| FIP-qPCR-F | ACTGCTGCAATGTTGTCCTGGAG |
| FIP-qPCR-R | GTCAATGCAGGCGAGGAGGT |
| HAKAI-qPCR-F | CCACCGTAATCAAGCCCGTC |
| HAKAI-qPCR-R | ATCCGCTCATCACATAGATAGCAG |
| MTA-qPCR-F | TTTCTCGATACTTGTCGTCACA |
| MTA-qPCR-R | CTTCAGAACAGTAATCAGCACG |
| ALKBH10B-qPCR-F | GAATCAACAATGGCCTATGGAC |
| ALKBH10B-qPCR-R | TTGAGAGAGAGTGTCAAAGGTC |
| ECT2-qPCR-F | GATCTCTCTGCAAACAAACCTG |

| | |
|---|---|
| ECT2-qPCR-R | GTTAAGGGTAGCCTGGTTAGTT |
| ECT3-qPCR-F | GGTATGGCTCTTACGGTTATGA |
| ECT3-qPCR-R | TGAGTTTCTCAGTCTCAGTCAC |
| ECT4-qPCR-F | CTAGCTCCAATTCGCATTACAC |
| ECT4-qPCR-R | CGTGCTACCATACTGACCATAT |
| UBQ10-qPCR-F | CACACTCCACTTGGTCTTGCGT |
| UBQ10-qPCR-R | TGGTCTTTCCGGTGAGAGTCTTCA |
| ACTIN-qPCR-F | GGTAACATTGTGCTCAGTGGTG |
| ACTIN-qPCR-R | CTCGGCCTTGGAGATCCACATC |
| HHL1-qPCR-F | GAAACATGAGAGCTGCACTTAG |
| HHL1-qPCR-R | CTTTCCGAATGACTCTTTTGCA |
| MPH1-qPCR-F | ACATCAACAAGTTCAAGCCTTC |
| MPH1-qPCR-R | GAGTTTCTGGCAGTGAAGTTTC |
| Deg1-qPCR-F | CTTTCTTCCTCCTCTGTACCTC |
| Deg1-qPCR-R | GTAAACAACGGAAGGAGTGTTC |
| cpTatC-qPVR-F | GTTCCAGTCATTCAGTTAC |
| cpTatC-qPCR-R | CCTACCACCACATATCTC |
| HCF243-qPCR-F | TTGCTGCTTATGATGTGTGAAC |
| HCF243-qPCR-R | TCCTGGTAAACATCTGACGAAA |
| HCF136-qPCR-F | TCACCTGATGGAAGATATGTCG |
| HCF136-qPCR-R | CAAACTCCTCTGTAATCCCAGT |
| HCF173-qPCR-F | GAGCAAACAGTATTTCGCTAGG |
| HCF173-qPCR-R | GCAGGCAAAGCTTTTATGTACT |
| HCF244-qPCR-F | CCGATGACTGAGACTAATAG |
| HCF244-qPCR-R | ATGTTGCTGAAGTAATCCT |
| TRX-M4-qPCR-F | AATCGTAGAACCAGAATCGCTC |
| TRX-M4-qPCR-R | TGCCATTCTGAATCAGACAGAT |
| Tha4-qPCR-F | CGTTGCTTTTTGGACCTAAGAA |
| Tha4-qPCR-R | TCTTTGTTACTCGTTGCTACCT |
| CPRabA5e-qPCR-F | GTAAAGAGGTCAAGGCTCAGAT |
| CPRabA5e-qPCR-F | TTCTAGATCACACTTGTTCCCC |
| FtsH1-qPCR-F | GTGGACTCTTCTTGCTTTTCAG |
| FtsH1-qPCR-R | TAGAACGTCCGAAATCCATAGG |
| FtsH2-qPCR-F | AAGAGAACAGACCCTCAATCAG |
| FtsH2-qPCR-R | CTCTATTGGTTGCAGCAACTAC |
| FtsH5-qPCR-F | AAATCGAAGTTTCAGGAAGTGC |
| FtsH5-qPCR-R | AATCTTAGCACCTAACGCAGTA |
| MET1-qPCR-F | ACTGGTGAGTTAACAGAGAAGG |
| MET1-qPCR-R | GCTTCTTCTGGTGTTGGTTTAG |
| CtpA1-qPCR-F | CGTTTAGCGAAGACAGGTTATG |
| CtpA1-qPCR-R | CATCTCCGTCAAGCCATAATTG |
| STN7-qPCR-F | CATTCCCATCCTTACGATCTGA |
| STN7-qPCR-F | TTCTTTGTCTTGCTTTGTACCG |
| STN8 -qPCR-F | TTAATCTGGAGATCAAGTC |

| | |
|---|---|
| STN8 -qPCR-R | TCTAGTATGCTCAAGTCT |
| 18s-F | GGTGCATGGCCGTTCTTA |
| 18s-R | CAGGCTGAGGTCTCGTTCAT |
| TUB2-F | ATCCGTGAAGAGTACCCAGAT |
| TUB2-R | AAGAACCATGCACTCATCAGC |
| Flag-tag Construct | |
| HHL1-flag-F | CTGGCGCGCCACTAGTATGGAAGTGAGTATGTCTTTGAATGC |
| A641, 661,670–G-flag-R | CGATGGATCCACTAGTGGCCTTGGCTTTCTCATCCGAGGCTTCTTCAGCTTTTTCGTCACTCCCCGGGCTCAGCGATGATA |
| A661–G-flag-R | CGATGGATCCACTAGTGGCCTTGGCTTTCTCATCCGAGGTTTCTTCAGCTTTTTCGTCACTCCCCGGGTTCAGCGATGATA |
| A641–G-flag-R | CGATGGATCCACTAGTGGCCTTGGCTTTCTCATCCGAGGTTTCTTCAGTTTTTTCGTCACTCCCCGGGCTCAGCGATGATA |
| A670–G-flag-R | CGATGGATCCACTAGTGGCCTTGGCTTTCTCATCCGAGGCTTCTTCAGTTTTTTCGTCACTCCCCGGGTTCAGCGATGATA |
| HHL1-flag-R | CGATGGATCCACTAGTGGCCTTGGCTTTCTCATCC |
| Promoter-GUS Construct | |
| VIR-Pro-F | CCATGATTACGAATTCGCCTGCCAGTTTCGGGCAAACC |
| VIR-Pro-R | GCAGGTCGACGGATCCCATGCCTCCAGGATTGATTGGTG |
| RNAi constructs | |
| VIR_RNAi1_F | TCGAGAAGGCTGCCTCCAATTTGTCAG |
| VIR_RNAi1_R | GATCCTGACAAATTGGAGGCAGCCTTC |
| VIR_RNAi2_F | AACTCGAGAAGTCCAGCCTTACTATCACC |
| VIR_RNAi2_R | GATCCTGACAAATTGGAGGCAGCCTTC |

**Supplementary Table 5. Specific interactors of VIR.**

| Protein accession | Gene | Short name |
|---|---|---|
| $m^6A$ writers | | |
| F4J8G7 | AT3G05680 | VIR |
| Q9LFC0 | AT5G01160 | HAKAI |
| Ribosome proteins | | |
| A8MQA1 | AT3G49010 | |
| A8MS28 | AT4G15000 | |
| B9DG17 | AT1G72370 | |
| F4IT48 | AT2G44120 | |
| O04603 | AT4G01310 | |
| Q2V4Q4 | AT1G07320 | |
| O65686 | AT4G34620 | |
| P17094 | AT1G43170 | |
| P25864 | AT3G44890 | |
| P34788 | AT1G22780 | |
| P38666 | AT3G53020 | |
| P46286 | AT2G18020 | |
| P49209 | AT1G33120 | |
| P49688 | AT2G41840 | |
| P51412 | AT1G35680 | |
| P51427 | AT3G11940 | |
| P60040 | AT2G01250 | |
| Q9FE65 | AT1G69620 | |
| Q93VC7 | AT5G30510 | |
| Q93VG5 | AT5G20290 | |
| Q93VI3 | AT1G27400 | |
| Q93VT9 | AT1G14320 | |
| Q9CAV0 | AT3G04840 | |
| Q9FLN4 | AT5G40950 | |
| Q9LF30 | AT5G15520 | |
| Q9FZ76 | AT1G18540 | |
| Q9LXG1 | AT5G15200 | |
| Q9LZ57 | AT5G02450 | |
| Q9LZH9 | AT3G62870 | |
| Q9SGA6 | AT3G02080 | |
| Q9SKX4 | AT2G43030 | |
| Q9T043 | AT4G27090 | |
| Regulators of RNA stability, processing, and translation | | |
| Q8VYJ4 | AT3G25150 | ATG3BP6 |
| F4K180 | AT5G26742 | RH3 |
| Q84W56 | AT5G63420 | RNJ |
| Q94BV4 | AT2G45810 | RH6 |
| Q9C5Z3 | AT3G57290 | eIF3e |

| | | |
|---|---|---|
| Q9LKA4 | AT3G15010 | UBA2C |
| Regulators of plastid gene expression | | |
| A0A178W0D3 | AT1G65260 | PTAC4 |
| A0A1P8B4I3 | AT4G13670 | PTAC5 |
| Q9STF2 | AT3G46780 | PTAC16 |
