# Supplementary material for: N6-methyladenosine RNA modification regulates photosynthesis during photodamage in plants
Source: Nat Commun. 2022 Dec 2;13:7441. doi: 10.1038/s41467-022-35146-z (PMC9718803; doi:10.1038/s41467-022-35146-z)
Supplement: Supplementary file 3 — Description of Additional Supplementary Files [file 41467_2022_35146_MOESM3_ESM.pdf]

Description of Additional Supplementary Files:

Supplementary Data 1. m^6A peaks in Col-0 seedlings under growth light conditions

Supplementary Data 2. m^6A peaks in Col-0 seedlings after 4h high light treatment

Supplementary Data 3. Differentially methylated m^6A peaks in Col-0 seedlings after 4h high light treatment

Supplementary Data 4. m^6A peaks in *vir-1* seedlings under growth light conditions

Supplementary Data 5. m^6A peaks in *vir-1* seedlings after 4h high light treatment

Supplementary Data 6. Differentially methylated m^6A peaks in *vir-1* compared to Col-0 seedlings under growth light conditions

Supplementary Data 7. Differentially methylated m^6A peaks in *vir-1* compared to Col-0 seedlings after 4h high light treatment

Supplementary Data 8. Differentially expressed genes in *vir-1* compared to Col0 seedlings under growth light conditions

Supplementary Data 9. Differentially expressed genes in *vir-1* compared to Col0 seedlings after 4h high light treatment

Supplementary Data 10. Differentially translated genes in *vir-1* compared to Col-0 seedlings under growth light conditions

Supplementary Data 11. Differentially translated genes in *vir-1* compared to Col-0 seedlings after 4h high light treatment

Supplementary Data 12. Differential TE genes in *vir-1* compared to Col-0 seedlings under growth light conditions

Supplementary Data 13. Differential TE genes in *vir-1* compared to Col-0 seedlings after 4h high light treatment

Supplementary Data 14. MS identified information
